# A Computational Framework Towards the Tele-Rehabilitation of Balance Control Skills

**DOI:** 10.3389/frobt.2021.648485

**Published:** 2021-06-09

**Authors:** Kubra Akbas, Carlotta Mummolo

**Affiliations:** Department of Biomedical Engineering, New Jersey Institute of Technology, Newark, NJ, United States

**Keywords:** telehealth, physical rehabilitation, balance assessment, margins of balance, balanced region, center of mass, balance perturbation, biped stability

## Abstract

Mobility has been one of the most impacted aspects of human life due to the spread of the COVID-19 pandemic. Home confinement, the lack of access to physical rehabilitation, and prolonged immobilization of COVID-19-positive patients within hospitals are three major factors that affected the mobility of the general population world-wide. Balance is one key indicator to monitor the possible movement disorders that may arise both during the COVID-19 pandemic and in the coming future post-COVID-19. A systematic quantification of the balance performance in the general population is essential for preventing the appearance and progression of certain diseases (e.g., cardiovascular, neurodegenerative, and musculoskeletal), as well as for assessing the therapeutic outcomes of prescribed physical exercises for elderly and pathological patients. Current research on clinical exercises and associated outcome measures of balance is still far from reaching a consensus on a “golden standard” practice. Moreover, patients are often reluctant or unable to follow prescribed exercises, because of overcrowded facilities, lack of reliable and safe transportation, or stay-at-home orders due to the current pandemic. A novel balance assessment methodology, in combination with a home-care technology, can overcome these limitations. This paper presents a computational framework for the in-home quantitative assessment of balance control skills. Novel outcome measures of balance performance are implemented in the design of rehabilitation exercises with customized and quantifiable training goals. Using this framework in conjunction with a portable technology, physicians can treat and diagnose patients remotely, with reduced time and costs and a highly customized approach. The methodology proposed in this research can support the development of innovative technologies for smart and connected home-care solutions for physical therapy rehabilitation.

## Introduction

The COVID-19 pandemic and subsequent stay-at-home orders put in place have caused a general reduction in physical mobility among countries across the globe (World Health Organization, 2020a; World Health Organization, 2020b; World Health Organization, 2020c). The fundamentally altered daily routine of the healthy young, adult, and elderly populations has been preventing them from performing the usual daily motor exercise. A direct effect of home confinement is the alteration of normal muscle activation during daily motion, which can cause muscular atrophy and other problems in motor function in otherwise healthy people of all ages. The negative impact that this reduction in mobility due to the COVID-19 pandemic has on muscles, neuromuscular junctions, and nerves has been especially stressed (Narici et al., 2020). As a secondary effect, the pandemic has made it particularly difficult for the pathological populations needing regular physical therapy and rehabilitation sessions to receive treatment. This can cause a deterioration of physical health in low-mobility patients, leading them to be more prone to falls and injuries (Visser et al., 2008; Levinger et al., 2017; Gandolfi et al., 2018). In addition, COVID-19 has also caused prolonged immobilization of patients within the hospital environment, leading researchers and medical professionals to brainstorm proper treatment protocols for these “secondary” mobility ailments (Iannaccone et al., 2020). The physical rehabilitation during this bedridden stage takes on passive and active modes, including resistance training and both static and dynamic balance training exercises (Iannaccone et al., 2020). In summary, sedentarism due to stay-at-home orders, lack of access to proper physical therapy, and prolonged immobilization during COVID-19-positive hospitalizations are the three main factors causing reduced mobility of various populations during COVID-19. These circumstances will continue to impact mobility in the medium/long term after the pandemic and motivate the need for alternative solutions for the delivery of physical therapy and rehabilitation in remote settings.

The use of telehealth and telerehabilitation can help counteract the above-mentioned challenges. Many benefits exist within switching to remote care: increased access to healthcare, reduction in overall cost, increased interaction with providers and patient engagement, the ability to provide both synchronous and asynchronous treatment, and the eventual generation of large datasets for broader scientific investigation and impact. Though many approaches to telemedicine currently exist (Ruiz et al., 2020; Seshadri et al., 2020), proper telemedicine for use in motor rehabilitation requires more functional components in combination with a computational framework that can systematically quantify specific aspects of motor performance, such as balance control skills.

Within the circumstances caused by the pandemic, there is a focus on restoring motor function in the following areas: deconditioning, strength, balance, and the ability to perform daily activities (Iannaccone et al., 2020). In particular, static and dynamic balance training must be performed to help restore the compromised postural stability due to the reduced exercise and exposure to proprioceptive stimuli (Iannaccone et al., 2020; World Health Organization, 2020b; World Health Organization, 2020c). Balance is influenced by many subsystems of the body (i.e., musculoskeletal, vestibular, ocular). This interconnectedness is why balance assessment within motor rehabilitation is critical to understanding the components of falling and how to prevent injury due to falls. Poor balance capabilities are among the leading causes for falls in the elderly (Visser et al., 2008; Levinger et al., 2017; Gandolfi et al., 2018), often resulting in limited mobility and reduced engagement in physical activities. Balance assessment methods are useful in helping practitioners determine the proper customized rehabilitation plan for their patients and allow researchers to develop better technology to conduct these assessments. Currently used methods range from subjective observations performed by medical professionals to more quantitative approaches, using medical devices specifically designed for computerized dynamic posturography (CDP) analysis. While many balance exercises are qualitatively designed and assessed in the clinical setting (e.g., Berg Balance Scale (Stevenson, 2001), Balance Error Scoring System (Bell et al., 2011), Activities Balance Confidence Scale (Raad et al., 2013), Y Excursion Balance Test (Kinzey and Armstrong, 1998), Star Excursion Balance Test (Glave et al., 2016)), the score subjectivity and variance across physical therapists can lead to inconsistencies in the rehabilitation outcomes. Furthermore, this qualitative approach is less feasible in a home-care setting, where the physical presence of a therapist is removed. Many clinics use CDP to determine a patient’s progress based on a quantitative type of assessment. For example, the NeuroCom SMART Balance Master can score a user’s performance through the sensory organization test of equilibrium and motor control test (Wagner et al., 2016). The sensory organization test evaluates postural stability under various sensory conditions, where the visual, proprioceptive, and vestibular senses are altered (Wagner et al., 2016; Olchowik and Czwalik, 2020). A final “equilibrium score” based on the center of gravity sway is associated to the sensory organization test to evaluate postural stability. Additionally, the motor control test uses a “latency score” to quantify the user’s postural response time in reaction to platform perturbations (Wagner et al., 2016).

Although the existing CDP devices are considered to be the best currently available technologies, these machines are too costly and substantial in size for in-home use, and they require a trained clinician to supervise the machine setup and operation. Technologies for home care rehabilitation must be portable, compact, and must have a user-friendly interface so that the general patient can operate the device with minimal training. Commercial balance training technologies aimed for the home environment are typically presented as “exergames” (exercise games), which utilize the body’s motion as a method of controlling gameplay and were developed to encourage activity through fun activities. For example, Wii Fit uses the body’s weight distribution on the balance board as a proxy indicator of balance (Wikstrom, 2012); the Kinect’s balance training games, based on body motion tracking, can provide a low-cost and accessible form of rehabilitation (Sápi et al., 2019); Neofect’s Smart Balance technology uses virtual environments and other visualizations to aid in stroke recovery by measuring center of pressure (COP), center of mass (COM), pressure distribution, and the traveled path during walking (Neofect, 2020); the Togu Challenge Disc made by MFT Bodyteamwork uses games and visual targets to train balance and tracks the general motion of the user (MFT Bodyteamwork, 2020); the Boditrak2 Balance Assessment System also uses games to assist with training and tracks balance through pressure mapping. These platforms have been proposed as portable solutions for increasing physical activity and for balance assessment and training (Kennedy et al., 2011; Goble et al., 2014; Sápi et al., 2019). However, they have limitations in both their technology (e.g., sensor quality, resolution, processing power) and assessment approach (e.g., simple tracking of body motion and pressure distribution as proxies for the evaluation of balance control). Research efforts are being made to develop more accurate portable and wearable technologies for quantitative balance assessment (Conforti et al., 2020; Torricelli et al., 2020) by including, for instance, inertial measurement units or electromyographic devices (Zampogna et al., 2020).

The limitations on the CDP and exergames technologies prevent simultaneous balance assessment and training from being properly performed at home. Specifically, existing assessment metrics and testing protocols need to be improved to better understand the mechanisms that affect postural control (Keshner and Fung, 2019). The theoretical/computational framework employed in any given technology to quantify rehabilitation outcomes must be both specific and comprehensive enough to capture the balance skills across multiple subjects and multiple exercises. At the same time, the systematic outcomes evaluation must not be too computationally intensive. Current balance assessments focus on selected specific measures, which provide only partial information on human balance control and may omit important components of balance related to the risk of falls (Sibley et al., 2015). Few specific indicators are typically captured in CDP or exergames (i.e., reaction time, movement velocity, endpoint excursion, COM and COP sway), whose deviation from a baseline only partially and indirectly characterizes the balance control ability of a subject (Chaudhry et al., 2004; Ganesan et al., 2015). Each of these mechanical indicators alone do not capture the state of balance of a system (i.e., whether the subject is balanced or not) nor do they characterize the overall capability of the subject to recover from general perturbations. As a result, the perspectives of quantification of human balance have not yet reached a golden standard (Sibley et al., 2015) and identifying a comprehensive set of quantifiable and customized targets for balance rehabilitation remains a challenge. Furthermore, the existing assessment metrics and technologies pose a limit to the type of movements that can be analyzed. In typical CDP protocols, movements are restricted to the device’s narrow platform and postural stability is assessed during periods of quiet standing (Glave et al., 2016). While numerous stability analyses have been proposed during general movements (e.g., sit-to-stand (Holmes et al., 2020), walking (Young et al., 2012), stair climbing (Herman et al., 2009), etc.), these have not been translated into a unified approach for the design of exercise protocols (and associated technology) involving multiple motor tasks. Assessment sessions typically analyze balance during the upright standing posture (postural stability) and tend to be independent from the physical therapy training sessions, which usually involve different types of dynamic motor tasks (Bayouk et al., 2006; Marioni et al., 2013; Levinger et al., 2017). For effective rehabilitation, assessment and training protocols should be simultaneously performed and combined into a unified technology-based framework for a broad range of balance exercises with quantifiable custom targets.

Recent studies have addressed the limited scope of quantification of existing balance assessment methods by addressing the stability of biped systems from a dynamic system perspective. In this context, balance is defined as the ability to maintain the state of a dynamic system inside a defined desired region of the state space (Pratt et al., 2017). The quantification of balance capabilities consists in the evaluation of a balanced region in the state space (Mummolo et al., 2017), also called basin of attraction or viability kernel (Aubin et al., 2011; Koolen et al., 2012; Zaytsev et al., 2015; Smith et al., 2017). The resulting balance stability criterion is a threshold that can discriminate between the conditions of balance and imbalance of a given biped system (Koolen et al., 2012; Mummolo et al., 2017; Koolen, 2019) by considering all possible factors that could lead to a loss of balance. These are more comprehensive approaches for monitoring the state of balance of a system and predicting fall, as opposed to tracking individual balance-related indicators. Furthermore, they can be generalized to various movements and translated into a broader range of static and dynamic exercise goals for simultaneous balance assessment and training.

In this study, a state-space balance criterion (Mummolo et al., 2017) is used to formulate metrics of balance that can serve as quantitative outcomes, as well as customized goals for the simultaneous assessment and training of balance control skills. This computational framework can be customized for a given patient in a rehabilitation regimen that involves multiple motor exercises. The evaluation of the proposed balance performance metrics relies on computational models of the human subject and associated balanced regions and requires capturing the subject’s COM motion and foot stance during the performed balance exercise. Based on these requirements, the proposed framework has the potential to be integrated with an affordable portable technology solution for customized tele-rehabilitation needs, in which the novel performance metrics can be evaluated on- and off-line to visually guide the patient during the balance exercises. The outcome of this research can contribute to tackling the issue of compromised mobility and motor performance of people living at the time of the COVID-19 pandemic.

## Simultaneous Balance Assessment and Training Method

A computational framework is proposed in which novel balance performance measures are formulated and implemented in exercises for simultaneous balance assessment and training. The use of the balance performance measures is twofold: 1) they are used as quantitative outcomes for general balance assessment and 2) they define customized and quantifiable balance training goals across multiple exercises. This novel rehabilitation paradigm requires the integration of the following components: 1) the theoretical formulation of the novel balance performance measures, 2) the design of exercises for which the balance performance is quantified, 3) a computationally tractable model of the user (human subject).

### Theoretical Formulation of Balance Performance Measures

A stability criterion based on the concept of balanced regions in the COM state space (Mummolo et al., 2017) is adopted in this study for the formulation of two categories of balance performance measures. This criterion uses nonlinear optimization for the numerical construction of a balance threshold in the state space of biped systems; it can be applied to general bipeds in various stance configurations, as well as to generic three-dimensional dynamic motor tasks.

**FIGURE 1:**
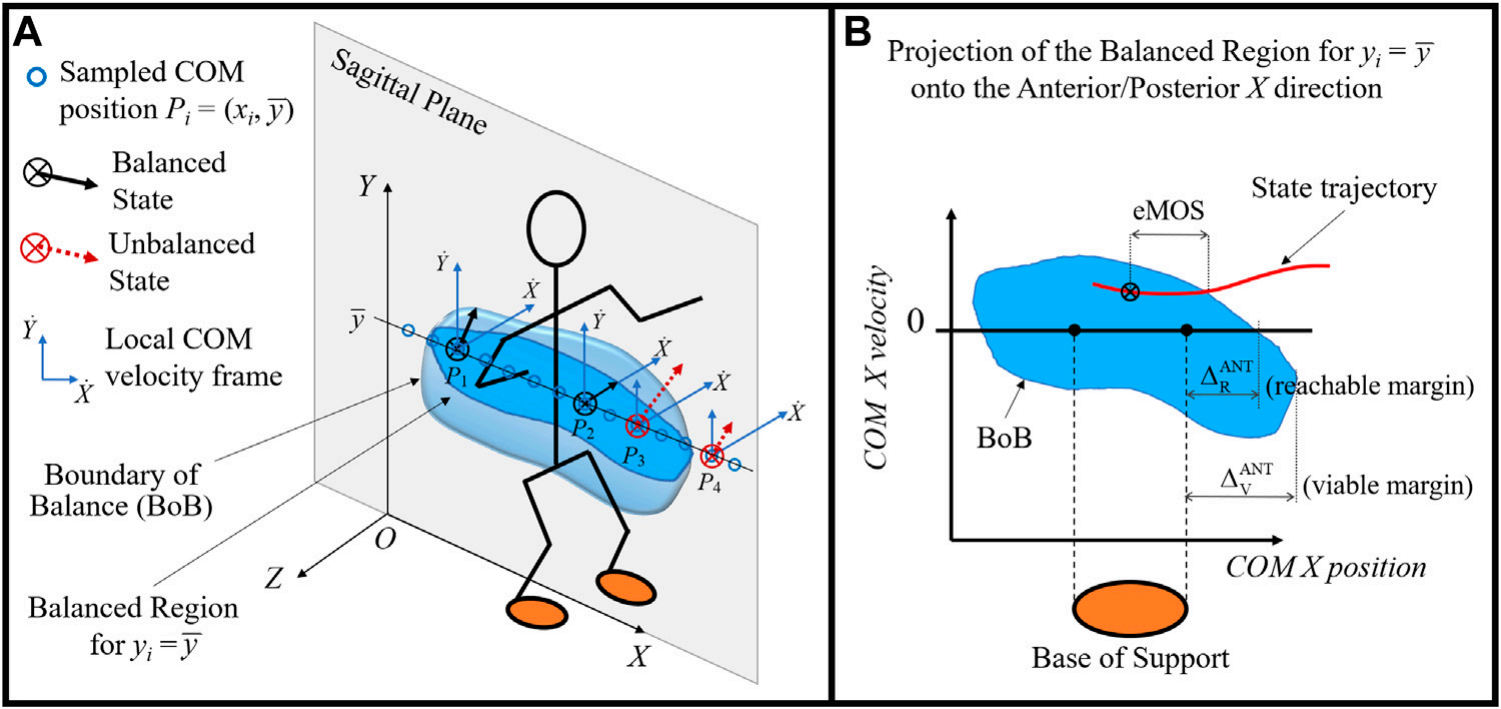
**(A)** Illustration of the balanced region concept (blue volume) and its boundary (BoB) in the sagittal plane of a biped system. At each sampled COM position (circles), the maximum feasible range of COM velocitiy perturbations are calculated and shown with respect to local velocity frames. Examples of balanced states at positions *P*_1_ and *P*_2_ and unbalanced states at positions *P*_3_ and *P*_4_ are shown, whose COM velocities fall inside and outside of the BoB, respectively. **(B)** The BoB is projected onto the (*X*, $\dot{X}$) plane to illustrate the concepts of viable and reachable boundary margins and their relationship with the base of support. The instantaneous state margin (eMOS) is also illustrated.

The *balanced region* is the set of all possible COM balanced states from which a given subject can reach an upright rest state, while avoiding a change in foot stance (Mummolo et al., 2017). The *balance stability criterion* states that a COM state located within the balanced region, i.e., balanced state, is the necessary condition for dynamic balance in generic biped models (Mummolo et al., 2017). A COM state outside of this region is defined as unbalanced and it predicts an inevitable change in foot stance at some time in the future. The boundary of the balanced region, called *boundary of balance* (BoB), represents the maximum limits of balance recovery of a subject while maintaining a given foot stance and is quantified in terms of maximum feasible range of COM velocity perturbations. The BoB is formed by the COM velocity extrema (minimum and maximum) calculated iteratively at various COM sampled positions, *P*_*i*_, *i* = 1, …, *N*, and along any specified direction; hence the balanced region is a partition of the six-dimensional state space of COM Cartesian position and velocity. For practical analysis and visualization, the BoB can be evaluated for a specified plane (e.g., sagittal plane) and projected onto a single direction of interest (e.g., anterior/posterior) (Figure 1).

The BoB is generated numerically by solving a series of constrained optimization problems. For each sampled COM initial position *P*_*i*_, optimization finds the limiting balance recovery trajectories in the joint space that drive the biped system from its extreme initial conditions (i.e., sampled COM initial position and minimum/maximum COM initial velocity) to a rest state, without a change in foot stance. The extremized COM initial state of each trajectory solution represents a point of the BoB. From any point of the BoB, there exists at least one controlled trajectory from which the subject can reach upright quiet stance without changing contacts. Alternatively, if the optimization finds no solution at a given *P*_*i*_, the feasible range of COM initial velocity that guarantees the COM will return to a stationary upright position without altering foot stance is null; in this case, any COM state at that specified *P*_*i*_ is unbalanced, i.e., outside of the BoB. The balancing trajectories generated from each point of the BoB satisfy the following constraints: 1) a final rest state (e.g., upright static posture), 2) various system and physics constraints (e.g., joint and torque limits, COP constraints), and 3) the preservation of the original stance (i.e., base of support). The resulting BoB is a stance-specific threshold that is customized using subject-specific body and joint parameters in the modeling, capturing the balance capabilities of a subject as predicted by the model. Details of the numerical optimization algorithm and its solution via sequential quadratic programming can be found in previous work (Mummolo et al., 2018b). The construction method of the balance threshold has been demonstrated for the study of gait and posture stability of human, robot, and exoskeleton systems (Mummolo et al., 2017; Mummolo et al., 2018a; Mummolo et al., 2018b; Mummolo and Vicentini, 2020; Mummolo et al., 2021).

In this study, the BoB is constructed point by point, by calculating the feasible range of COM velocity at various COM positions *P*_*i*_ sampled in the sagittal plane at a selected COM height, i.e., *P*_*i*_ = (*x*_*i*_, $\bar{y}$) (Figure 1A). The COM initial velocity is extremized along the anterior/posterior direction of interest (*X*) and the BoB results into the following set of limiting COM initial conditions in the sagittal plane: (*x*_*i*_, $\bar{y}$, ${\dot{x}}_{i,\lim}^{\text{ANT/POS}}$, ${\dot{y}}_{i}^{\text{ANT/POS}}$), for *i* = 1−*N*. The three-dimensional BoB can then be projected onto the state space of COM *X* position and velocity (Figure 1B) for practical analysis.

Two categories of balance performance metrics are formulated in the COM state space, based on the balanced region concept described above:

1) *Boundary Margins* are numerical indicators that characterize the dimension of a balance region relative to the base of support (Figure 1B). These indicators include the reachable ($\Delta_{R}$) and viable ($\Delta_{V}$) boundary margins, which quantify the capability of a subject in a given stance to recover from internal and external perturbations, respectively, along a specified direction. Similar to the balanced region, both boundary margins do not depend on a specific motor task but are a property of the selected models for both the subject’s body and the desired stance configuration.

The reachable boundary margin $\Delta_{R}$ is the distance between the point of the BoB with zero velocity and the edge of the base of support, measured in both anterior and posterior directions. It predicts how far the body can displace its COM outside of the footprint and then invert its motion (hence, zero velocity) to recover balance without any external impulse or change of contact. This margin identifies a limit to the amount of self-induced perturbations (i.e., internal) that a subject can sustain from a given stance; hence it is analogous to a maximum voluntary COM sway in dynamic conditions (i.e., out of the base of support (Mummolo et al., 2013)).

The viable boundary margin $\Delta_{V}$ is the distance between the point of the BoB with maximum COM position and the edge of the base of support, measured in both anterior and posterior directions. It quantifies the range of COM positions outside of the footprint for which a feasible COM velocity exists. The balanced states included in between the reachable and the viable margins cannot be attained through the body internal dynamics alone, but they are viable initial conditions resulting from an external impulse. Therefore, the viable margin identifies a limit to the amount of externally induced perturbations that a subject can sustain while in a given stance.

2) *State Margins* are numerical indicators that characterize the instantaneous state of balance for a given trajectory, by measuring its relative distance to the BoB. Depending on how this distance is measured in the state space, these indicators can include position, velocity, or mixed margins. In this study, the extended Margin of Stability (eMOS) (Mummolo et al., 2021) is used as a position margin that quantifies the distance from a given state to the BoB along the position coordinate of the state space (Figure 1B). The eMOS is equivalent to the Margin of Stability (MOS) for a linear inverted pendulum (LIP) model (Hof et al., 2005; Mummolo et al., 2021), but it can be applied to any generic biped system. This indicator, unlike the boundary margin, is specific to the motor task performed by the subject in a given stance, allowing for the continuous evaluation of the COM state of balance.

### Design of Balance Assessment and Training Exercises

The application of the balanced region and balance performance measures within the rehabilitation context is presented. The two categories of balance performance measures are used as design criteria for rehabilitation exercises in which balance performance is simultaneously quantified and trained. For a given subject and foot stance, the balanced region and boundary margins predicted by the optimization-based algorithm provide a reference map for defining customized target states across multiple exercises.

**FIGURE 2:**
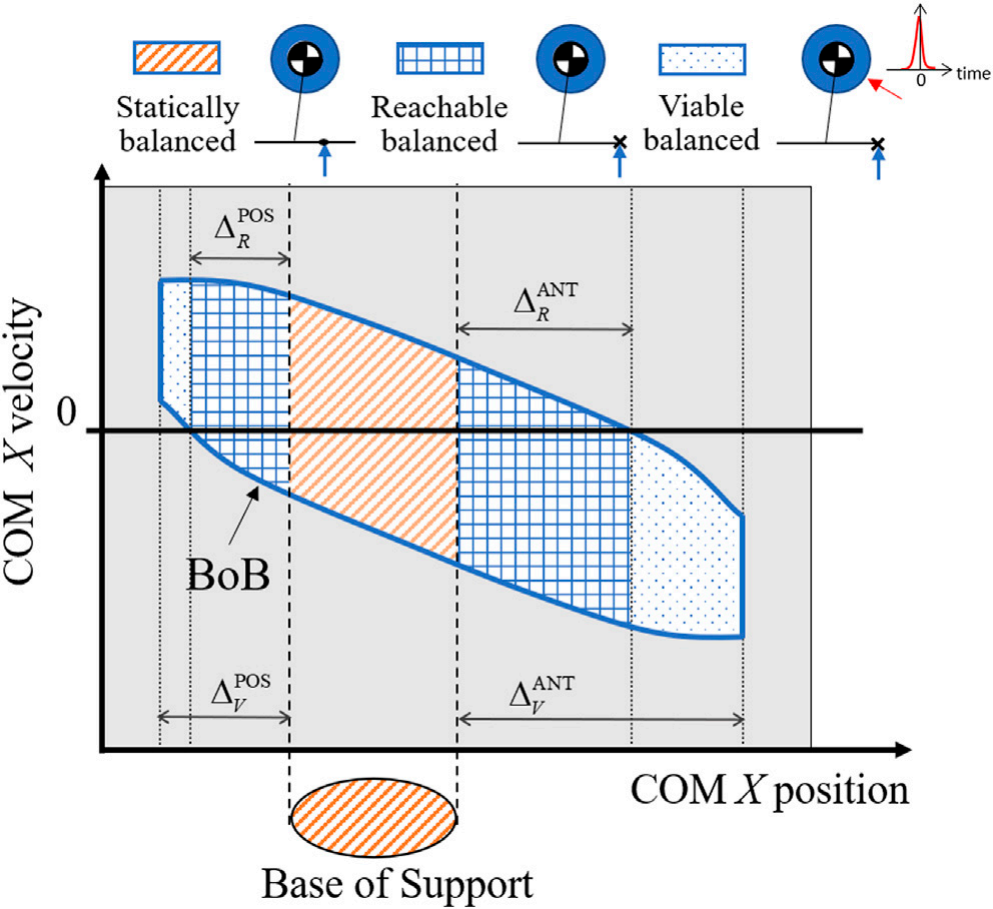
The reachable and viable boundary margins in both the anterior (ANT) and posterior (POS) directions divide the balanced region into three partitions, each identifying a type of balanced states: statically, reachable, and viable balanced states. For each type of balanced state, an illustration of the corresponding balance control strategy is shown using a simple legged system with a point mass, flywheel, and foot link.

The intersections of the BoB with the edges of the base of support and the boundary margins in both anterior and posterior directions identify three partitions of the balanced region (Figure 2):

1) The portion of the balanced region characterized by a COM ground projection within the edges of the base of support (Mummolo et al., 2013) is the set of statically balanced states: a state in this partition can be driven to a static equilibrium configuration by controlling the COP position within the given base of support and/or through the regulation of whole-body linear and angular momentum. From a statically balanced state the motion could in theory be stopped instantaneously without causing the system to lose balance.
2) The portion of balanced region included within the reachable margins is the set of reachable balanced states: each of these states has a COM position outside of the footprint, hence is dynamically balanced. Because this partition contains both positive and negative COM velocity, the COM can enter it from a statically balanced state using internal dynamics and then invert its motion to recover balance. Balance recovery from reachable balanced states is only possible through the rotation of multiple body segments about the COM that generates a stabilizing angular momentum and its derivative, similar to the effect of a flywheel. Within this partition, the COP cannot be controlled and balancing must rely on a combination of favorable (i.e., balanced) COM initial conditions and whole-body inertial effects over a finite interval of time.
3) The portion of balanced region characterized by COM positions that are outside of the reachable margins, but within the viable margins, is the set of viable balanced states: similar to the reachable states, they are also dynamically balanced and must rely on favorable initial conditions and whole-body inertial effects in order to reach a static equilibrium within a finite interval of time. The difference from reachable states is that the system’s COM can enter this partition only through externally imposed perturbations, e.g., external impulse. However, once the COM state is inside this partition (i.e., it becomes viable) the external push can cease and the system in the given stance can recover balance by means of its initial conditions and actuation capacity.

The above partition-based analysis of the balanced region provides a reference map that characterizes the different stability nature and means of control for a COM state within each partition (Figure 2). Statically, reachable, and viable balanced states are three categories of exercises targets that can be assigned to the subject’s COM during a rehabilitation exercise. The amount of sustainable perturbations and recovery strategy associated with each target category is known a priori; this constitutes a novel approach to the design of balance exercise as compared to traditional balance perturbation experiments, in which there is no a priori knowledge of the effects of a given perturbation on the COM stability, hence no clear and meaningful balance target can be established.

Two types of balance exercises (perturbation-based and motor task-based) are proposed in which the requirements of a desired user’s motion are imposed in terms of COM initial, target, and final states. For each exercise, the final state is a statically balanced state, while the initial and target states are assigned to the different partitions of the balanced region, based on the exercise desired outcome. In addition, each exercise has a prescribed foot contact (or sequence of contacts), used to evaluate the associated contact-dependent balanced region.

**FIGURE 3:**
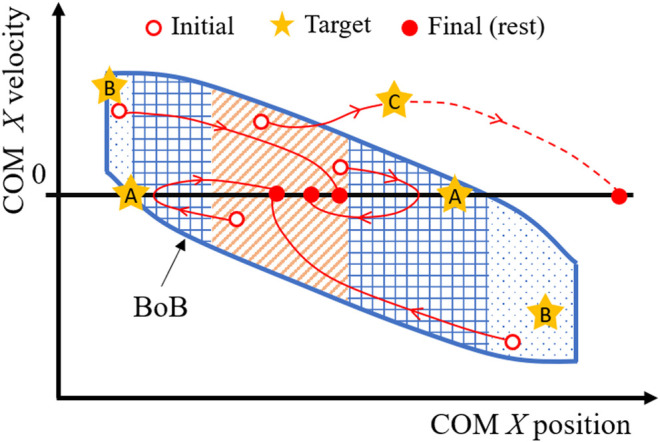
Design of motor task for balance exercises (perturbation-based and motor task-based), where the COM is guided through initial, target, and final states. Targets A are selected progressively close to the reachable boundary margin, to find the experimental maximum COM sway of a given subject, i.e., the capacity of withstanding internal perturbations. Targets B are selected progressively close to the viable boundary margin, to find the experimental limits of external perturbations that a subject can withstand. Targets C are selected to drive a motor task at a known distance either inside or outside of the BoB and determine in real time the instantaneous state margin throughout the motion.

The first type of balance exercise consists in perturbation experiments guided by target states placed progressively closed to the boundaries of the balanced region (i.e., the BoB) (Targets A and B, Figure 3). This exercise has the purpose of determining the amount of internal and external perturbations that can be attained by the subject in experimental conditions (i.e., experimental reachable and viable margins), where internal perturbations are the impulses generated by the subject when initiating or performing a movement, whereas external perturbations require the impulse generated through contact with another object, such as pushing off from a wall or the ground. The experimental boundary margins are then compared with the exercise targets, i.e., the margins predicted by the simulated numerical boundary (i.e., numerical boundary margins).

Two examples of perturbation-based balance exercises during standing posture are described:

1) Example training exercise to reject internal perturbations from upright stance—Starting from rest, the subject is asked to initiate a forward/backward motion, come as close as possible to reachable Target A, and then invert its motion to reach a final rest state, all while maintaining a double stance configuration. Experimental reachable margins resulting from this exercise are quantified as the maximum anterior/posterior position reached by the subject’s COM before inverting its motion. The experimental and numerical reachable margins are compared to have a relative measure of the subject’s maximal COM sway capacity along a specific direction.
2) Example training exercise to reject external perturbations from upright stance—Starting from rest, the subject is asked to perform a pre-balancing task in which the COM should attain initial conditions as close as possible to viable Target B using the external impulse generated, for instance, by a hand-push on a fixed handle. The subject’s state at the end of the push-off motion is recorded as the initial viable state of the balancing motion, which will terminate at upright equilibrium with no change in foot stance. The most extreme viable initial state that can be successfully attained by the subject gives the experimental viable margin, which is compared with the numerical counterpart to have a relative measure of the subject’s limits of recovery from external perturbations.

This first type of perturbation-based exercises aims at simultaneously quantifying and training the general perturbation rejection capability of a subject relative to a given stance. Using the reference map, specific portions of the balanced region can be targeted for a given patient, to enhance a particular type and direction of balance control.

The second type of balance exercise is specific for a motor task and focuses on quantifying the balance performance of a specific trajectory. Multiple target states are assigned either inside or outside (e.g., Target C, Figure 3) of the BoB, as via-points of a desired motor task placed at selected distances from the boundary as quantified by the state margin eMOS. Throughout the exercise, the state margin is also utilized to quantify a subject’s instantaneous level of balance/imbalance. As the subject’s COM state trajectory remains within the boundary (i.e., balanced), the resulting eMOS values are positive, while states that exit the boundary result in negative eMOS values, leading to an inevitable foot contact change in the future. Given the general applicability of the BoB, the motor tasks for this type of exercise can be selected among common daily lives activities, including standing, frontal and lateral stepping, walking, and sit-to-stand actions.

In summary, the boundary and state margins are used in the proposed exercises as balance targets relative to the overall subject’s balance capabilities quantified by the balanced regions. At the same time, the experimental boundary/state margins are evaluated as balance performance outcomes of a given exercise and compared to the respective numerical values predicted by the model, to assess a patient’s relative level of balance performance in a given stance and during a specific motor task, respectively.

When implementing these exercises within tele-rehabilitation settings, the desired and current COM state and foot stance information must be recorded and visualized by the patient. The patient’s COM motion can be captured by existing methods (Lafond et al., 2004) and will be visually guided by targets prescribed within the different partitions of a balanced region (i.e., statically balanced, reachable, and viable). Meanwhile the COM state and foot stance information can be displayed as an overlay on the subject’s balanced region. This would allow the patient and the physician to receive visual feedback on their balance performance during the exercises, in which a change in foot stance and/or a COM state crossing the BoB will signal an unbalanced motion. Additionally, the physician (remotely) could adjust the initial, final, and target states with respect to the balanced region maps, according to the patient’s training status and needs.

### Human Subject Modeling Approach

The theoretical/computational framework described above can be applied to any generic human body model, ranging from whole-body (Mummolo et al., 2019) to reduced-order (Mummolo et al., 2015b) biped mechanisms, and to various contact configurations between the feet and the environment (Mummolo et al., 2018b). The balance criterion and performance indicators can therefore be implemented in a broad range of balance rehabilitation protocols, including static, dynamic, and multi-stance exercises.

**FIGURE 4:**
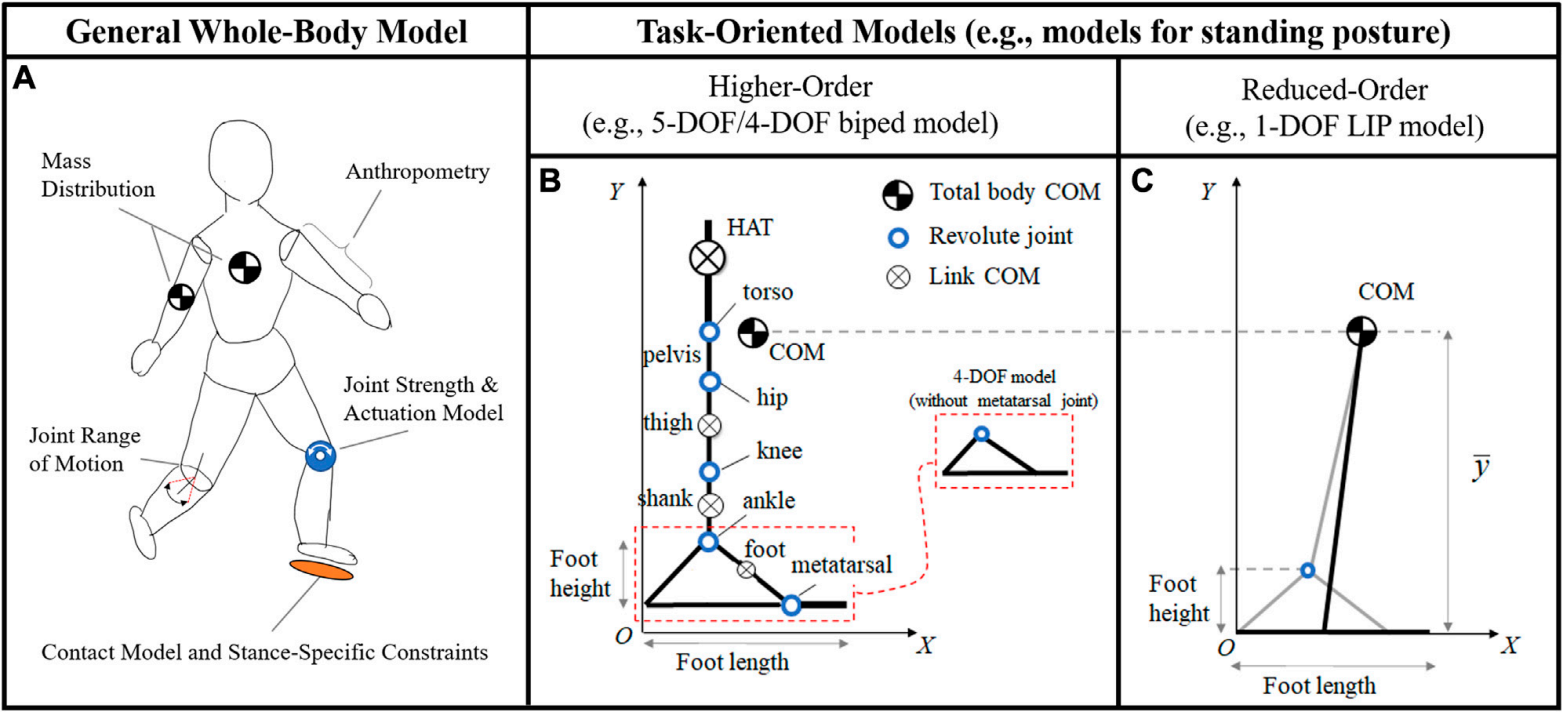
**(A)** General human body models take into consideration individual anthropomorphic parameters, joint strengths and actuations, mass distributions, range of motions, ground contact modeling, and stance-specific constraints. Whole-body models are valid for the representation of any general motor task, although their simulation can be computationally intractable. In practice, task-oriented models can give a practical computation of the balanced region for the specific task considered. For balance stability during standing posture, both higher- and reduced-order models in the sagittal plane can be established. The higher-order models **(B)** include more detailed subject-specific parameters at the link and joint level, upper and lower body segments, as well as multi-segment feet (5-DOF model), as described in previous work (Mummolo and Vicentini, 2020). The LIP model **(C)** constrains the motion of the COM at a fixed height $\bar{y}$, equal to the *Y*-coordinate of the body’s COM while standing and does not include joint-level design parameters.

The construction of a subject’s BoB via the optimization-based algorithm previously described requires the establishment of the dynamic model of the subject’s body. Links’ length and mass distribution, joint strengths and range of motions, ground contact modeling, and stance-specific constraints are specified and the subject’s dynamics can then be described using common robotic modeling approaches for floating-base robotic systems with multiple degrees-of-freedom (DOF) (Figure 4A). The dynamics and stability of systems in multi-contact stances is usually more challenging to describe (Del Prete et al., 2018; Orsolino et al., 2020), given the indeterminacy in the system’s foot reactions when the legs form a closed loop with the ground (Mummolo et al., 2015a). Different modeling choices (e.g., number of DOF, single vs. double stance, planar vs. three-dimensional) lead to different BoB and balanced regions. The complexity of the established biped model should reflect a good balance between the accuracy in the numerical prediction of the subject’s balanced region and the computational performance of the BoB algorithm. In practice, when a balance region is sought for a specific motor task, a task-oriented modeling approach can be pursued for higher computational efficiency. Depending on the motor task requirements of a given exercise (e.g., range of desired COM displacement, anatomical plane and direction of interest, and expected foot contacts), the simplest model that fits those criteria should be selected.

In this study, balance exercises during standing posture are considered for demonstration purposes, which are characterized by a symmetric double stance, small variation of COM height, and significant COM perturbations in the sagittal plane along the anterior/posterior direction. Three increasingly complex models of human body that satisfy the exercise requirements are implemented in the balance assessment framework: a 1-DOF linear inverted pendulum (LIP) model, with a single mass and a flat foot (Figure 4C); a 4-DOF model with upper and lower body segments and a rigid foot (i.e., without metatarsal joint; Figure 4B); a 5-DOF model with upper and lower body segments and a two-link foot (i.e., with metatarsal joint; Figure 4B). All three models are in the sagittal plane and are reasonable candidates to analyze dynamic balance in the anterior-posterior direction. The balanced regions and their margins provide a systematic approach to evaluate the effects of each modeling assumption on the predictive capability of the model’s balance stability. An accurate model would result in a balanced region that encompasses all experimental COM state trajectories resulting from the exercises in which balance is preserved.

## Demonstrative Results and Discussion

The novel paradigm for simultaneous balance assessment and training is demonstrated with the results of balanced regions and balance performance measures calculated for different models of human standing posture. Experimental balance recovery trajectories extracted from published literature (Patton et al., 1999) are used to exemplify the proposed perturbation-based and motor task-based exercises and associated margins calculation.

### Nondimensional Balanced Regions and Boundary Margins for Standing Posture

**FIGURE 5:**
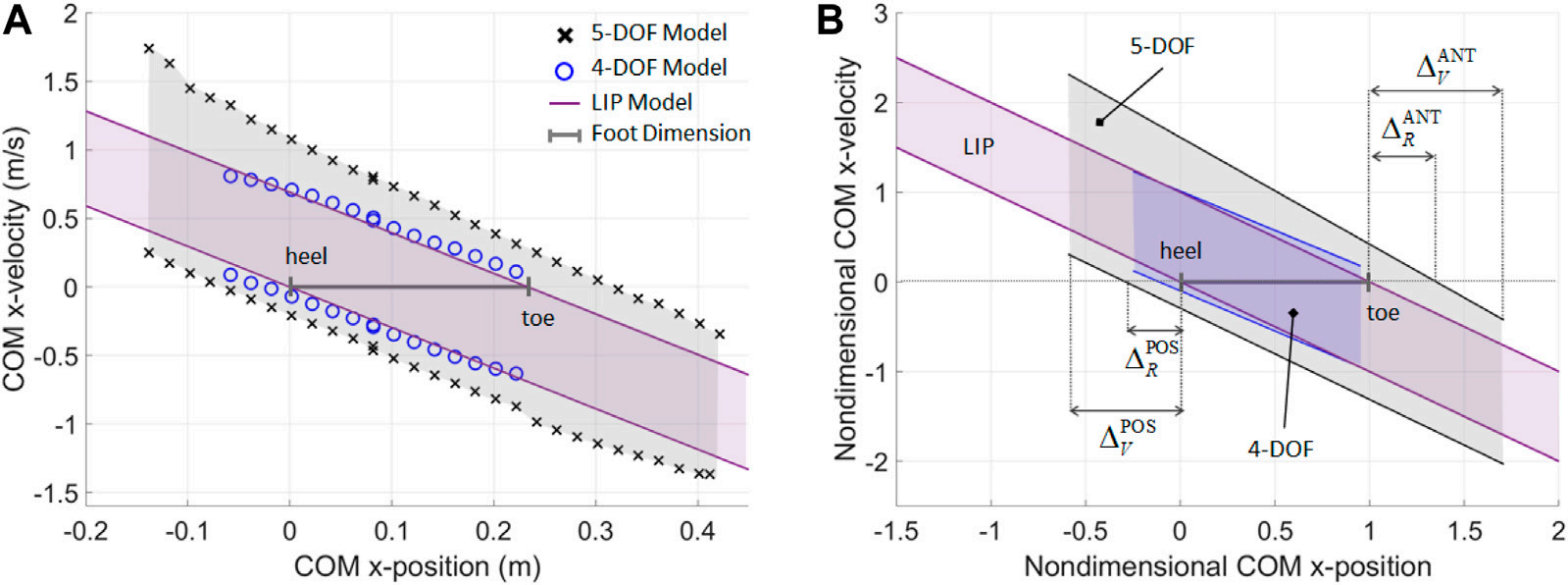
**(A)** Numerical construction of the BoB for a reference subject in upright stance, when the subject’s body is modeled with a 5-DOF mechanism with a two-link foot, a 4-DOF mechanism with a rigid foot, and a 1-DOF LIP with flat foot. **(B)** Nondimensional fitted lines provide a general model of BoB for the three types of biped mechanism considered (*R* ^2^ > 0.99). The nondimensional upper and lower BoB limits are $-x\leq \dot{x}\leq -x+1$ (LIP), $-0.89x-0.099\leq \dot{x}\leq -0.88x+1.01$ (4-DOF), and $-1.02x-0.29\leq \dot{x}\leq -1.19x+1.61$ (5-DOF).

The balanced region results are presented for three increasingly complex models of a human subject in the sagittal plane, i.e., the LIP, 4-DOF, and 5-DOF models, to illustrate the effects of body and foot modeling choices on the predicted range of allowable perturbations during standing posture (Figure 5A). The anthropometric parameters and joint angle/torque limits of the reference subject are from the literature (Winter 2005; Mummolo and Vicentini, 2020). The LIP and the 4-DOF models have a rigid foot link with no metatarsal joint, which is assumed to maintain a fixed contact with the ground at all times; the 5-DOF model includes a two-link foot, where a metatarsal joint and a multimodal foot-ground interaction model (Mummolo et al., 2021) allow the foot to rotate about its heel and metatarsal. All models have a total foot length *fl* = 0.23 m.

For the higher-order models, the BoB is numerically constructed using the proposed algorithm. The COM velocity extrema are calculated along the anterior (+*X*) and posterior (−*X*) direction, by sampling the COM initial positions at a constant height $\bar{y}$ = 1.12 m, corresponding to the subject’s COM *Y*-coordinate in the upright standing configuration. The BoB of the LIP model can be found analytically using the linear inequalities that limit the position of the extrapolated center of mass ($XCoM=x+\dot{x}/\omega$) within the base of support [0, *fl*] (Mummolo et al., 2021), i.e., $-\omega x\leq \dot{x}\leq -\omega x+fl\omega$, where x and $\dot{x}$ are the COM position and velocity, respectively, $\bar{y}$ and $\omega =\sqrt{g/\bar{y}}$ are the pendulum’s length and natural frequency.

Fitted models of the BoB and enclosed balanced regions are obtained in the nondimensional COM state space for each of the three biped models considered (Figure 5B), where the COM position and velocity are normalized with respect to *fl* and flω, respectively. The nondimensional formulation of the BoB provides a general characterization of the balanced regions for upright standing posture in three different subject modeling approaches. These nondimensional linear models can used for multiple individuals, with different anthropometric parameters, when adopted in a home-care rehabilitation context.

The three balanced regions are representative of the different balance control strategies that can be employed by each biped model. This is demonstrated quantitatively through the calculation of the nondimensional boundary margins (Table 1), which give a relative measure of maximum balanced COM displacement as a percentage of foot size. The only means of balance control for the LIP model is the regulation of the COP within the limits of its flat foot; as a result, the LIP reachable boundary margins are zero, indicating that the COM sway cannot exceed the base of support in order to preserve balance, according to this reduced-order model predictions. In addition, the linear inequalities for the *XCoM* do not provide limits to the range of feasible COM positions, therefore the LIP viable margins are undefined. On the other hand, the higher-order models show a greater range of sustainable COM velocity perturbation for a given COM position and along both anterior and posterior directions. The 4-DOF and 5-DOF models have posterior reachable boundary margins equal to 11 and 28.8% of the foot size, respectively, predicting that the COM sway can exceed the rear edge of the base of support while retaining the ability to invert its motion thanks to angular momentum inertial effects. The posterior viable margins in both higher-order models quantify the range of viable negative COM positions at which an external impulse can be applied to stabilize the system. The anterior reachable margin for the 5-DOF model predicts that the COM sway can exceed the front edge of the base of support by 35.2% of foot size, and then recover balance. The negative values for the anterior boundary margins in the 4-DOF model indicate that the set of reachable and viable balanced states is null in the anterior direction, due to the kinematic restrictions of a rigid foot, which do not allow significant COM displacement at the given COM height $\bar{y}$.

**TABLE 1:** Anterior (ANT) and posterior (POS) nondimensional boundary margins of the general linear models of BoB calculated for the reduced- and higher-order body models.

	*Reachable boundary margin*		*Viable boundary margin*	
	$\Delta_{R}^{\text{POS}}$	$\Delta_{R}^{\text{ANT}}$	$\Delta_{V}^{\text{POS}}$	$\Delta_{V}^{\text{ANT}}$
5-DOF MODEL two-link foot	0.288	0.352	0.591	0.808
4-DOF MODEL rigid foot	0.110	−0.047	0.248	−0.047
LIP MODEL rigid foot	0.0	0.0	n.a.	n.a.

The greater perturbation rejection capability predicted by the higher-order models is due to the multiple balancing strategies that can be employed by a multi-DOF system (e.g., ankle, hip, upper-body, and general angular momentum regulation) in addition to COP control. In particular, the largest boundary margins are for the 5-DOF model, due to the presence of a two-link foot that enables the additional balancing strategy of heel-to-toe foot rocking, which increases the range of feasible COM positions and velocity perturbations.

The above boundary margins values predicted by the three models of human posture can be used as both targets and outcomes in COM perturbation experiments in tele-rehabilitation settings, to simultaneously assess and train a subject’s overall balance performance ability to reject internal and external perturbations.

### Use of Boundary and State Margins during Balance Exercises

To showcase the role of boundary margins in a balance rehabilitation exercise, empirical data relative to push recovery exercises published in the literature (Patton et al., 1999) is partially extracted and adapted to the proposed framework. In the experiments of the reference study (Patton et al., 1999), subjects were asked to pull on a horizontal handle, targeting various percentages of their maximum pulling force in order to attain perturbed COM initial conditions in the posterior direction. At the end of the pull, the balancing trajectories of the subjects’ COM as they recovered balance while standing on two feet were recorded. It should be noted that, although derived from a different study, the experimental data illustrated is the result of perturbation-based balancing exercises analogous to those proposed in this framework, with the difference that the experiments in (Patton et al., 1999) are guided by force-based targets, while the proposed experiments would be conducted using the viable and reachable margins as novel balance-related targets.

**FIGURE 6:**
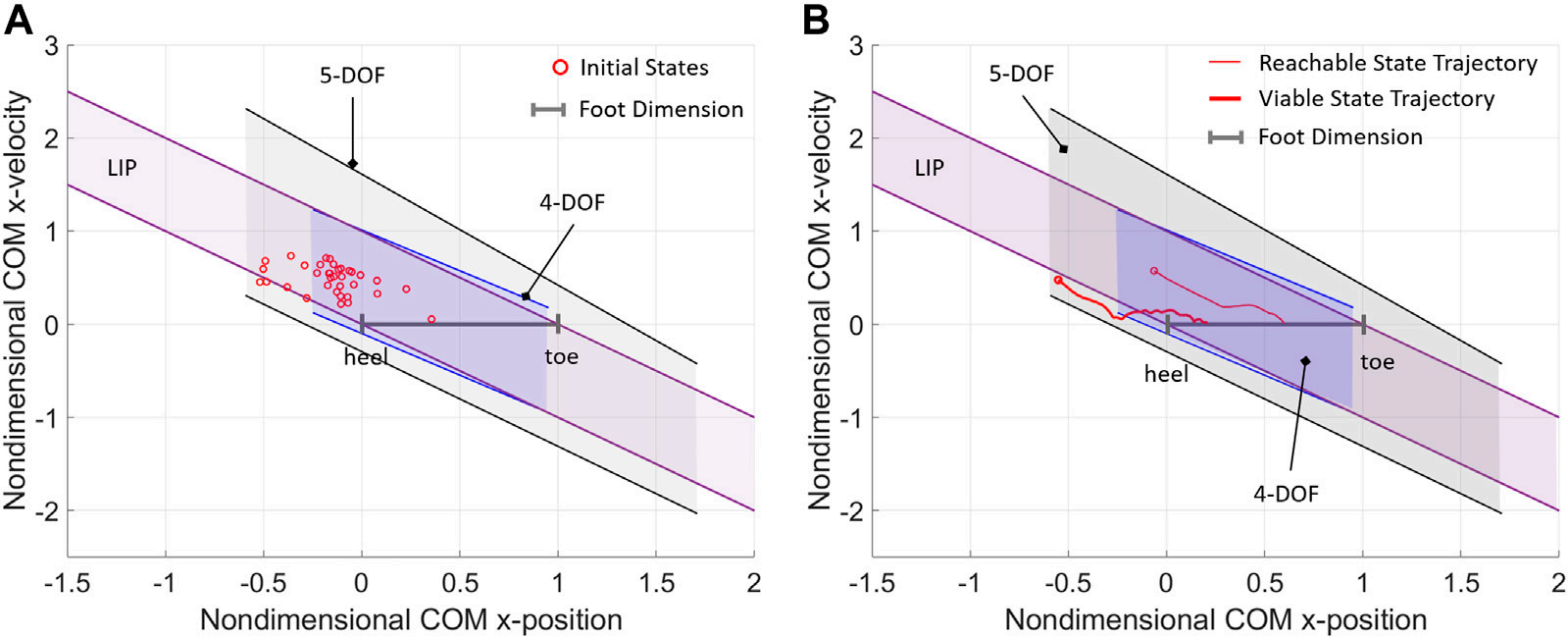
**(A)** A collection of initial states of a subject during externally-imposed perturbation experiments (Patton et al., 1999), compared with the nondimensional models of balanced region for standing posture. **(B)** Two experimental balance recovery trajectories extracted from (Patton et al., 1999), where the subject standing on two feet recovers balance and reaches static equilibrium after receiving an external impulse the subject standing on two feet recovers balance and reaches static equilibrium after receiving an external impulse.

Here, 35 perturbed COM states for one subject of the reference study are extracted, normalized, and represented against the nondimensional balanced regions (Figure 6A). The most extreme COM initial state is used to estimate the subject’s experimental viable margin in the posterior direction (0.519), which is closely predicted by the posterior viable margin of the 5-DOF model (0.591). The 4-DOF posterior viable margin fails to enclose eight highly dynamic initial states, hence the model underestimates the subject’s balance performance in terms of rejection of external perturbations. Four initial states are either outside or on the boundary of the LIP balanced region; while this indicates that the reduced order model predicts balance in about 88% of the selected initial states, it should be noted that the LIP analytical boundaries did not provide quantifiable target viable states. These results demonstrate how the balance performance of a subject can be assessed and trained based on perturbation experiments and the evaluation of experimental boundary margins. In the current results and reference study, no data is available to demonstrate the experimental evaluation of reachable margins, which is left to future work.

Lastly, the evaluation of balance performance in the motor task- based exercise is illustrated by two example trajectories extracted from the reference study (Patton et al., 1999), which exemplify the analysis of a generic balancing trajectory with respect to the balanced regions of the subject (Figure 6B). In both experimental trajectories, the subject was able to recover upright static equilibrium without altering the double foot stance. The reachable balanced trajectory starts from initial conditions within the reachable boundary margins of the higher-order models and well within the LIP balanced region. The first half of the reachable balanced trajectory starts from a dynamic reachable state (with COM outside of the base of support) and reaches an upright statically stable state (with COM approximately aligned with the ankle joint); this segment of trajectory appears close to the linear passive dynamics of the LIP, suggesting that the first part of the balancing motion may rely mostly on the favorable initial conditions, while angular momentum effects may not be relevant. Conversely, the viable balanced trajectory starts from initial conditions outside of all reachable boundary margins and even outside of the LIP and 4-DOF balanced regions; however, the initial state is viable with respect to the boundary margins predicted by the 5-DOF model, which is an indication that the balancing motion must rely on multiple strategies, including the angular momentum and foot rocking strategy, in addition to the favorable initial conditions. These results suggest that for such a highly dynamic balancing motion, a higher-order mechanism with a multi-segment foot model gives a better prediction of an individual’s balance performance, as compared to biped models with lower DOF and rigid foot.

**FIGURE 7:**
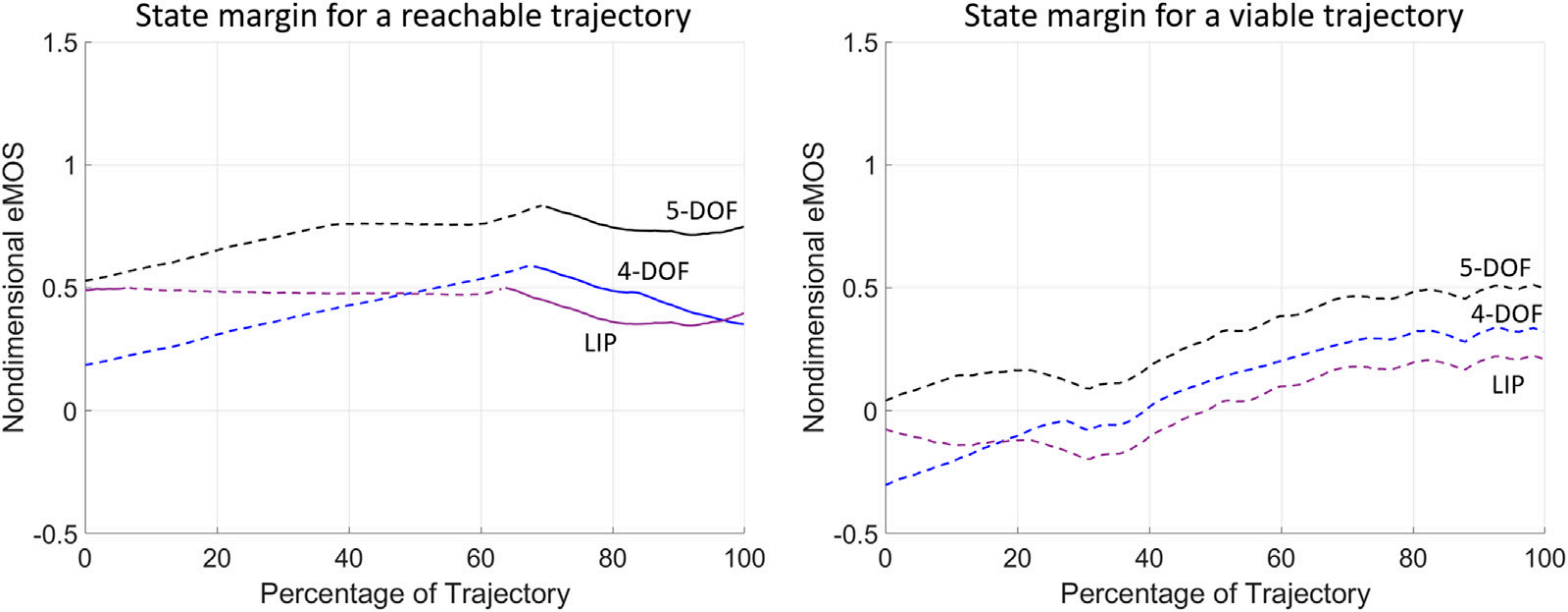
The instantaneous state margin (eMOS) is calculated throughout the balancing motion of the two example trajectories from (Patton et al., 1999). The eMOS values are calculated for the normalized trajectories and BoB of each biped model considered, where the solid and dashed lines correspond to state margins relative to the upper and lower BoB lines, respectively.

The state margin eMOS is calculated for the two example trajectories to evaluate their instantaneous level of balance or imbalance (Figure 7). The nondimensional eMOS quantifies the distance from a given state of the trajectory with respect to both the upper and lower bounds of each balance threshold, measured along the position coordinate; here, the smallest distance from either lower (dashed lines, Figure 7) and upper (solid lines, Figure 7) bounds is shown, since it represents the most critical balance condition. A positive eMOS value indicates that the trajectory is within a balanced region, where a greater eMOS absolute value corresponds to a greater balance safety margin, while a smaller eMOS absolute value indicates a closer proximity to the unbalanced region; the opposite is true for negative eMOS. When the LIP is used the eMOS coincides with the MOS (Mummolo et al., 2021), and its positive values range from 0 to 1.

The eMOS of the reachable trajectory indicate that all three biped models correctly predict a balanced trajectory that is closer to the lower BoB boundaries for approximately 65% of the motion, after which the COM state results closer to the upper BoB lines (Figure 7; left). Throughout the reachable trajectory, the BoB of the 4-DOF and LIP models underestimate the instantaneous margin of balance, as compared to the 5-DOF model, hence predicting a smaller level of balance throughout the motion. The eMOS of the viable trajectory indicates that only the 5-DOF model correctly predict a balanced motion, with a state margin always closer to the lower BoB limits (Figure 7; right). The 4-DOF and LIP models present negative eMOS values at the beginning of the viable trajectory, which would wrongly predict the subject’s inability to recover balance from those initial conditions, without external help and without changing foot stance.

The above analysis of balanced regions, boundary and state margins demonstrates an objective method of assessing a patient’s progress throughout treatment. The nondimensional boundary margins have highlighted the differences between the different modeling approaches of human standing posture: the inclusion of a multi-segment foot can lead to a more accurate balance characterization in real human subjects. Boundary margins allow the selection of customized and quantifiable targets for training balance recovery from internal and external perturbations, while state margins provide a numerical benchmark of the subject’s balance capabilities during a particular trajectory. All proof-of-concept results demonstrate the benefits of having a balance criterion that can be extended to higher-order models that can more accurately predict dynamic stability, as compared with reduced-order models.

## Conclusions and Future Work

This study proposed a novel balance training and assessment computational technique, illustrated through an example subject performing a postural stability exercises obtained from a reference study (Patton et al., 1999). By using the balanced regions as reference maps, new balance exercises can also be developed for the furthering of current physical rehabilitation approaches. The application of the proposed framework to home-care rehabilitation, which is essential during and after the COVID-19 pandemic, is briefly discussed. The balance performance indicators are proposed as both targets and outcomes of balance exercises that require only tracking and visual feedback of desired vs. current COM motion and foot stance, as opposed to, for example, the measurement of COP, ground reactions, and external impulse forces profiles, which may not be easily integrated into an affordable and portable device. For this reason, the proposed theoretical/computational framework could be a promising initial step for the development of innovative devices for the remote assessment and rehabilitation of balance performance in patients affected by reduced mobility. The integration of the presented criterion for quantitative balance assessment with a portable instrumented platform would contribute to the advancement of postural stability analysis in three ways: first, it would allow patients and the general population to participate in highly customized in-home physical therapy treatment plans to prevent or treat mobility disorders while also being systematically evaluated; second, it could open the way for clinicians to design and test balance exercises that can include dynamic stance changes and other general motor tasks; third, it has the capability to generate a unified benchmarking dataset of significant volume across multiple populations (e.g., of different ages and pathological conditions), which would boost further investigation on the medium/long term effects of COVID-19 on people’s balance ability and the associated fall risk.

## Data Availability

The raw data supporting the conclusions of this article can be made available by the authors, upon request.

## References

[B1] Aubin J. P. Bayen A. M. Saint-Pierre P. (2011). Viability Theory: New Directions. Springer-Verlag Berlin Heidelberg: Springer Science & Business Media.

[B2] Bayouk J.-F. Boucher J. P. Leroux A. (2006). Balance Training Following Stroke: Effects of Task-Oriented Exercises With and Without Altered Sensory Input. Int. J. Rehabil. Res. 29 (1), 51–59. 10.1097/01.mrr.0000192100.67425.84 16432390

[B3] Bell D. R. Guskiewicz K. M. Clark M. A. Padua D. A. (2011). Systematic Review of the Balance Error Scoring System. Sports Health 3 (3), 287–295. 10.1177/1941738111403122 23016020 PMC3445164

[B7] Chaudhry H. Findley T. Quigley K. S. Bukiet B. Ji Z. Sims T. (2004). Measures of Postural Stability. J. Rehabil. Res. Dev. 41 (5), 713–720. 10.1682/jrrd.2003.09.0140 15558401

[B8] Conforti I. Mileti I. Del Prete Z. Palermo E. (2020). Measuring Biomechanical Risk in Lifting Load Tasks Through Wearable System and Machine-Learning Approach. Sensors 20 (6), 1557. 10.3390/s20061557 PMC7146543 32168844

[B9] Del Prete A. Tonneau S. Mansard N. (2018). Zero Step Capturability for Legged Robots in Multicontact. IEEE Trans. Robot. 34 (4), 1021–1034. 10.1109/tro.2018.2820687

[B10] Gandolfi M. Geroin C. Picelli A. Smania N. Bartolo M. (2018). Assessment of Balance Disorders, Advanced Technologies for the Rehabilitation of Gait and Balance Disorders. Cham, Switzerland: Springer, 47–67.

[B11] Ganesan M. Kanekar N. Aruin A. S. (2015). Direction-Specific Impairments of Limits of Stability in Individuals With Multiple Sclerosis. Ann. Phys. Rehabil. Med. 58 (3), 145–150. 10.1016/j.rehab.2015.04.002 25960358

[B12] Glave A. P. Didier J. J. Weatherwax J. Browning S. J. Fiaud V. (2016). Testing Postural Stability: Are the Star Excursion Balance Test and Biodex Balance System Limits of Stability Tests Consistent? Gait Posture 43, 225–227. 10.1016/j.gaitpost.2015.09.028 26514832

[B13] Goble D. J. Cone B. L. Fling B. W. (2014). Using the Wii Fit as a Tool for Balance Assessment and Neurorehabilitation: The First Half Decade of "Wii-Search". J. Neuroeng. Rehabil. 11, 12. 10.1186/1743-0003-11-12 24507245 PMC3922272

[B14] Herman T. Inbar-Borovsky N. Brozgol M. Giladi N. Hausdorff J. M. (2009). The Dynamic Gait Index in Healthy Older Adults: The Role of Stair Climbing, Fear of Falling and Gender. Gait Posture 29 (2), 237–241. 10.1016/j.gaitpost.2008.08.013 18845439 PMC2709498

[B15] Hof A. L. Gazendam M. G. J. Sinke W. E. (2005). The Condition for Dynamic Stability. J. Biomech. 38 (1), 1–8. 10.1016/j.jbiomech.2004.03.025 15519333

[B16] Holmes P. D. Danforth S. M. Fu X.-Y. Moore T. Y. Vasudevan R. (2020). Characterizing the Limits of Human Stability During Motion: Perturbative Experiment Validates a Model-Based Approach for the Sit-to-Stand Task. R. Soc. Open Sci. 7 (1), 191410. 10.1098/rsos.191410 32218959 PMC7029948

[B17] Iannaccone S. Castellazzi P. Tettamanti A. Houdayer E. Brugliera L. de Blasio F. (2020). Role of Rehabilitation Department for Adult Covid-19 Patients: The Experience of the San Raffaele Hospital of Milan. Arch. Phys. Med. Rehabil. 101 (9), 1656–1661. 10.1016/j.apmr.2020.05.015 32505489 PMC7272153

[B18] Kennedy M. W. Schmiedeler J. P. Crowell C. R. Villano M. Striegel A. D. Kuitse J. (2011). “Enhanced Feedback in Balance Rehabilitation Using the Nintendo Wii Balance Board,” in IEEE 13th International Conference on e-Health Networking, Applications and Services, June 13–15, 2011, (Columbia MO: IEEE), 162–168.

[B19] Keshner E. A. Fung J. (2019). Editorial: Current State of Postural Research - Beyond Automatic Behavior. Front. Neurol. 10, 1160. 10.3389/fneur.2019.01160 31736864 PMC6834784

[B20] Kinzey S. J. Armstrong C. W. (1998). The Reliability of the Star-Excursion Test in Assessing Dynamic Balance. J. Orthop. Sports Phys. Ther. 27 (5), 356–360. 10.2519/jospt.1998.27.5.356 9580895

[B21] Koolen F. A. (2019). Balance Control and Locomotion Planning for Humanoid Robots Using Nonlinear Centroidal Models. Doctoral dissertation. Cambridge MA: Massachusetts Institute of Technology.

[B22] Koolen T. De Boer T. Rebula J. Goswami A. Pratt J. (2012). Capturability-Based Analysis and Control of Legged Locomotion, Part 1: Theory and Application to Three Simple Gait Models. Int. J. Robot. Res. 31 (9), 1094–1113. 10.1177/0278364912452673

[B23] Lafond D. Duarte M. Prince F. (2004). Comparison of Three Methods to Estimate the Center of Mass during Balance Assessment. J. Biomech. 37 (9), 1421–1426. 10.1016/s0021-9290(03)00251-3 15275850

[B24] Levinger P. Dunn J. Bifera N. Butson M. Elias G. Hill K. D. (2017). High-Speed Resistance Training and Balance Training for People With Knee Osteoarthritis to Reduce Falls Risk: Study Protocol for a Pilot Randomized Controlled Trial. Trials 18 (1), 1–11. 10.1186/s13063-017-2129-7 28821271 PMC5563024

[B25] Marioni G. Fermo S. Zanon D. Broi N. Staffieri A. (2013). Early Rehabilitation for Unilateral Peripheral Vestibular Disorders: A Prospective, Randomized Investigation Using Computerized Posturography. Eur. Arch. Otorhinolaryngol. 270 (2), 425–435. 10.1007/s00405-012-1944-4 22310838

[B26] MFT Bodyteamwork (2020). MFT Challenge Disc. Available at: https://www.challenge-disc.com/en/ (Accessed April 10, 2021).

[B27] Mummolo C. Akbas K. Carbone G. (2021). State-Space Characterization of Balance Capabilities in Biped Systems With Segmented Feet. Front. Robot. AI 8, 613038. 10.3389/frobt.2021.613038 33718440 PMC7952635

[B28] Mummolo C. Mangialardi L. Kim J. H. (2015a). “Concurrent Contact Planning and Trajectory Optimization in One Step Walking Motion,” in ASME 2015 International Design Engineering Technical Conferences and Computers and Information in Engineering Conference, August 2–5, 2015, Boston, MA: American Society of Mechanical Engineers Digital Collection.

[B29] Mummolo C. Mangialardi L. Kim J. H. (2015b). “Identification of Balanced States for Multi-Segmental Legged Robots Using Reduced-Order Model,” in IEEE-RAS 15th International Conference on Humanoid Robots (Humanoids), November 3–5, 2015, (Seoul, Korea: IEEE), 914–919.

[B30] Mummolo C. Mangialardi L. Kim J. H. (2013). Quantifying Dynamic Characteristics of Human Walking for Comprehensive Gait Cycle. J. Biomech. Eng. 135 (9), 91006. 10.1115/1.4024755 23775488

[B31] Mummolo C. Mangialardi L. Kim J. H. (2017). Numerical Estimation of Balanced and Falling States for Constrained Legged Systems. J. Nonlinear Sci. 27 (4), 1291–1323. 10.1007/s00332-016-9353-2

[B32] Mummolo C. Peng W. Z. Agarwal S. Griffin R. Neuhaus P. D. Kim J. H. (2018a). Stability of Mina V2 for Robot-Assisted Balance and Locomotion. Front. Neurorobot. 12, 62. 10.3389/fnbot.2018.00062 30374298 PMC6196256

[B33] Mummolo C. Peng W. Z. Gonzalez C. Kim J. H. (2018b). Contact-Dependent Balance Stability of Biped Robots. J. Mech. Robot. 10 (2), 021009. 10.1115/1.4038978

[B34] Mummolo C. Peng W. Z. Kim J. H. (2019). “Whole-Body Balancing Criteria for Biped Robots in Sagittal Plane,” in International Design Engineering Technical Conferences and Computers and Information in Engineering Conference, August 18–21, 2019, Anaheim, CA: American Society of Mechanical Engineers (ASME).

[B35] Mummolo C. Vicentini G. (2020). “Limits of Dynamic Postural Stability with a Segmented Foot Model,” In: Editors Ateshian G. Myers K. Tavares J. Computer Methods, Imaging and Visualization in Biomechanics and Biomedical Engineering. CMBBE 2019. Lecture Notes in Computational Vision and Biomechanics. 36, (Cham, Switzerland: Springer), 256–270. 10.1007/978-3-030-43195-2_21

[B36] Narici M. De Vito G. Franchi M. Paoli A. Moro T. Marcolin G. (2020). Impact of Sedentarism Due to the COVID-19 Home Confinement on Neuromuscular, Cardiovascular and Metabolic Health: Physiological and Pathophysiological Implications and Recommendations for Physical and Nutritional Countermeasures. Eur. J. Sport Sci. 21 (4), 614–635. 10.1080/17461391.2020.1761076 32394816

[B37] Neofect (2020). Smart Balance. Available at: https://www.neofect.com/us/smart-balance (Accessed April 10, 2021).

[B38] Olchowik G. Czwalik A. (2020). Effects of Soccer Training on Body Balance in Young Female Athletes Assessed Using Computerized Dynamic Posturography. Appl. Sci. 10 (3), 1003. 10.3390/app10031003

[B39] Orsolino R. Focchi M. Caron S. Raiola G. Barasuol V. Caldwell D. G. (2020). Feasible Region: An Actuation-Aware Extension of the Support Region. IEEE Trans. Robot. 36 (4), 1239–1255. 10.1109/tro.2020.2983318

[B40] Patton J. L. Pai Y.-C. Lee W. A. (1999). Evaluation of a Model that Determines the Stability Limits of Dynamic Balance. Gait Posture 9 (1), 38–49. 10.1016/s0966-6362(98)00037-x 10575069

[B41] Pratt J. Ott C. Hyon S. H. Goswami A. Vadakkepat P. (2017). “Introduction to Humanoid Balance,” in Humanoid Robotics: A Reference. Editors Goswami A. Vadakkepat P. (Dordrecht, Netherlands: Springer). 1315–1321.

[B42] Raad J. Moore J. Hamby J. Rivadelo R. L. Straube D. (2013). A Brief Review of the Activities-Specific Balance Confidence Scale in Older Adults. Arch. Phys. Med. Rehabil. 94 (7), 1426–1427. 10.1016/j.apmr.2013.05.002

[B43] Ruiz I. Contreras J. Garcia J. (2020). Towards a Physical Rehabilitation System Using a Telemedicine Approach. Comput. Methods Biomech. Biomed. Eng. Imaging Vis. 8 (6), 671–680. 10.1080/21681163.2020.1795929

[B44] Sápi M. Domján A. Fehérné Kiss A. Pintér S. (2019). Is Kinect Training Superior to Conventional Balance Training for Healthy Older Adults to Improve Postural Control?. Games Health J. 8 (1), 41–48. 10.1089/g4h.2018.0027 30153062

[B45] Seshadri D. R. Davies E. V. Harlow E. R. Hsu J. J. Knighton S. C. Walker T. A. (2020). Wearable Sensors for COVID-19: A Call to Action to Harness Our Digital Infrastructure for Remote Patient Monitoring and Virtual Assessments. Front. Digit. Health 2, 8. 10.3389/fdgth.2020.00008 PMC8521919 34713021

[B46] Sibley K. M. Beauchamp M. K. Van Ooteghem K. Straus S. E. Jaglal S. B. (2015). Using the Systems Framework for Postural Control to Analyze the Components of Balance Evaluated in Standardized Balance Measures: A Scoping Review. Arch. Phys. Med. Rehabil. 96 (1), 122–132. 10.1016/j.apmr.2014.06.021 25073007

[B47] Smith V. A. Lockhart T. E. Spano M. L. (2017). Basins of Attraction in Human Balance. Eur. Phys. J. Spec. Top. 226 (15), 3315–3324. 10.1140/epjst/e2016-60345-4 29629019 PMC5886352

[B48] Stevenson T. J. (2001). Detecting Change in Patients With Stroke Using the Berg Balance Scale. Aust. J. Physiother. 47 (1), 29–38. 10.1016/s0004-9514(14)60296-8 11552860

[B49] Torricelli D. Rodriguez-Guerrero C. Veneman J. F. Crea S. Briem K. Lenggenhager B. (2020). Benchmarking Wearable Robots: Challenges and Recommendations From Functional, User Experience, and Methodological Perspectives. Front. Robot. AI 7, 168. 10.3389/frobt.2020.561774 PMC7805816 33501326

[B50] Visser J. E. Carpenter M. G. van der Kooij H. Bloem B. R. (2008). The Clinical Utility of Posturography. Clin. Neurophysiol. 119 (11), 2424–2436. 10.1016/j.clinph.2008.07.220 18789756

[B51] Wagner D. R. Saunders S. Robertson B. Davis J. E. (2016). Normobaric Hypoxia Effects on Balance Measured by Computerized Dynamic Posturography. High Alt. Med. Biol. 17 (3), 222–227. 10.1089/ham.2016.0040 27689470

[B53] Wikstrom E. A. (2012). Validity and Reliability of Nintendo Wii Fit Balance Scores. J. Athl. Train. 47 (3), 306–313. 10.4085/1062-6050-47.3.16 22892412 PMC3392161

[B54] Winter D. A. (2005). Biomechanics and Motor Control of Human Movement. 3rd ed. New York, NY: Wiley.

[B56] World Health Organization (2020b). #BeActive for the UN International Day of Sport for Development and Peace. Available at: https://www.who.int/news-room/detail/06-04-2020-beactive-for-the-un-international-day-of-sport-for-development-and-peace (Accessed July 27, 2020).

[B57] World Health Organization (2020c). #HealthyAtHome - Physical Activity. Available at: https://www.who.int/news-room/campaigns/connecting-the-world-to-combat-coronavirus/healthyathome/healthyathome---physical-activity (Accessed September 6, 2020).

[B58] World Health Organization (2020a). Coronavirus. Available at: https://www.who.int/health-topics/coronavirus (Accessed September 26, 2020).

[B59] Young P. M. M. Wilken J. M. Dingwell J. B. (2012). Dynamic Margins of Stability During Human Walking in Destabilizing Environments. J. Biomech. 45 (6), 1053–1059. 10.1016/j.jbiomech.2011.12.027 22326059 PMC3321251

[B60] Zampogna A. Mileti I. Palermo E. Celletti C. Paoloni M. Manoni A. (2020). Fifteen Years of Wireless Sensors for Balance Assessment in Neurological Disorders. Sensors 20 (11), 3247. 10.3390/s20113247 PMC7308812 32517315

[B61] Zaytsev P. Hasaneini S. J. Ruina A. (2015). “Two Steps is Enough: No Need to Plan Far Ahead for Walking Balance,” in IEEE International Conference on Robotics and Automation (ICRA), 26-30 May 2015, (Seattle, WA: IEEE), 6295–6300.

